# Evaluating Vocal Cord Dysfunction in Infants Following the Norwood Operation: A National Pediatric Cardiology Quality Improvement Collaborative Analysis

**DOI:** 10.1007/s00246-025-03919-0

**Published:** 2025-06-15

**Authors:** Liza Hashim, Alicia Chaves, Mark Conaway, Jeffrey Vergales

**Affiliations:** 1 Division of Cardiology, Department of Pediatrics, University of Virginia Health Systems, Charlottesville, VA USA; 2 Division of Cardiology, Department of Pediatrics, University of Maryland School of Medicine, Baltimore, MD USA; 3 Department of Public Health Sciences, University of Virginia, Charlottesville, VA USA; 4 Pediatric Cardiology, UVA Medical Center, 1215 Lee Street, Charlottesville, VA 22903 USA

**Keywords:** Norwood, Single ventricle, Vocal cord dysfunction

## Abstract

The Norwood operation is associated with postoperative complications including vocal cord dysfunction (VCD). This multicenter study evaluates VCD prevalence, risk factors, and diagnostic practices in a high-risk population. We performed a retrospective review of data from the National Pediatric Cardiology Quality Improvement Collaborative registry, examining infants who underwent the Norwood operation (S1P) with a surgical shunt and either completed stage 2 palliation or died after S1P discharge. The primary outcome was the endoscopic diagnosis of VCD after Norwood operation. Analysis of patient characteristics, operative details, and postoperative outcomes was performed to identify associations with VCD. Center variation in VCD was also assessed. Among the 2342 infants, VCD was diagnosed in 21.7%. Aortic arch reintervention (AAR) occurred in 19% and was associated with higher odds of VCD (OR 1.525, *p* = 0.001). Infants with VCD had higher rates of G-tube placement (42% vs. 22%,* p* < 0.001), though tracheostomy was uncommon (3.3%). Center analysis revealed lower rates of VCD at higher volume centers. Over time, AAR rates declined while VCD diagnoses increased. Our findings suggest that the Norwood operation plays a significant role in the development of VCD. The link between AAR and VCD is complex but may be influenced by institutional differences in evaluation practices. Given its impact on feeding and potential for persistence, VCD represents an important postoperative complication. Comprehensive postoperative care, low threshold to screen for VCD, and a multidisciplinary approach to management is needed to continually improve outcomes in infants undergoing the Norwood operation.

## Introduction

The Norwood operation is the first of three staged palliative surgeries performed in infants with hypoplastic left heart syndrome and variants with aortic hypoplasia. Although modern surgical techniques have improved mortality, postoperative complications still affect short and long-term outcomes in this population. Vocal cord dysfunction (VCD) is a known complication following congenital heart surgery, including the Norwood operation, with reported incidence rates ranging widely from 1 to 59% [1, 2]. This can result from iatrogenic injury during surgery to the recurrent laryngeal nerve (RLN) which innervates the intrinsic muscles of the larynx and is essential for sound production, swallowing mechanics, and mobility of vocal folds during breathing. Injury to the RLN leads to vocal cord immobility, manifesting clinically as dysphonia, feeding challenges, and respiratory distress [3]. In severe cases, it may require non-cardiac procedures like gastrostomy tube placement or tracheostomy [4, 5]. Diagnosis is variable, typically being made by direct visualization of vocal cords with direct fiberoptic laryngoscopy (DFL), fiberoptic endoscopic evaluation of swallowing (FEES), or by laryngeal ultrasound [1, 3, 4, 6, 7].

Patient factors such as prematurity, genetic syndromes, and congenital airway anomalies have been identified as risk factors for the development of VCD [3, 8, 9]. Much of what is known about this critical subject is derived from single-center studies, with considerable variability in reported rates. This impedes gaining a deeper understanding of VCD and inhibits identifying targets for improvement. This study aims to determine the prevalence of and risk factors for VCD in patients who have undergone the Norwood operation and to explore diagnostic practice variations after the Norwood operation among infants enrolled in a large multicenter database.

## Methods

### Study Design

We performed a retrospective analysis of longitudinal data in the National Pediatric Cardiology Quality Improvement Collaborative (NPC-QIC) registry. The NPC-QIC is a multicenter, international network that safeguards data on infants with hypoplastic left heart syndrome and other single ventricle variants. Phase I of data collection focused on improving outcomes during the interstage period in infants undergoing staged palliation. Phase II, launched in August 2016, expanded these efforts by evaluating patient outcomes from time of diagnosis through their first birthday. Phase II data consisted of prenatal and postnatal information and important operative and postoperative details following stage 1 palliation (S1P) and stage 2 palliation (S2P). For the purposes of this study, only Phase II data were reviewed as it was more complete up through S2P. The study period included data collected between August 2016 and February 2023, with individual patient follow-up concluding at 1 year of age. All data were de-identified by the central registry prior to review.

The study cohort included all infants who first underwent Norwood operation and either underwent S2P with bidirectional Glenn or died after S1P discharge. Since VCD has been related to iatrogenic injury to RLN during aortic arch reconstruction, infants who underwent a hybrid approach (pulmonary artery band with or without PDA stent) and Damus-Kaye-Stansel connection with surgical shunts but without arch reconstruction were not reviewed. VCD was reviewed if it was diagnosed at any time after the Norwood operation, including interstage and after S2P operation as exact date of diagnosis could not be determined from the registry. Infants who died after S1P discharge were included as they were equally as likely to have had postoperative evaluation of VCD as the cohort who survived to S2P.

Infants without S2P data and those without clearly documented death following S1P discharge were excluded due to the various potential reasons for the missing fields. They may have been lost to follow-up, transferred care, withdrawn from the study, or died. Since the temporal relationship between missing data and the opportunity for VCD evaluation could not be reliably established, this exclusion was applied. Infants with large amounts of missing data were also excluded for the above reason. Infants who died during hospitalization immediately following index operation were excluded as they were not equally as likely to have been diagnosed with VCD. The University of Virginia Institutional Review Board acknowledged that this question was not subject to its oversight.

### Variables

The primary outcome of this study was the diagnosis of VCD following the Norwood operation. The NPC-QIC records VCD as a postoperative complication following the S1P and S2P surgeries. It is defined as the presence of poor or no vocal cord movement diagnosed via endoscopy, with or without clinical findings or stridor, hoarse voice or cry. Secondary predictor variables included patient specific and perioperative risk factors that would contribute to the diagnosis of VCD. The relationship between center volume for Norwood operation and VCD diagnosis rates was examined to determine if the volume of Norwood operations performed influences the rate of VCD diagnoses. Center volume for Norwood was defined as the total number of *eligible* Norwood operations (from the total cohort) performed at each center over the study period (August 2016 to February 2023). Center volume was the average (or mean) number of Norwood procedures per year. It only counts the years where the hospital contributed data. While this was not perfect as it was difficult to distinguish between hospitals with 0 procedures and hospitals not participating in the database that year, this metric allowed us to compare centers that started contributing data in different years. In center-level analyses, each center is represented once.

We also included birth year in the temporal analysis to evaluate trends in VCD and AAR diagnoses over time. Since existing literature on this did not suggest clinical relevance, further analysis was not conducted. Additionally, the study aimed to evaluate postoperative complications including non-cardiac reoperations and rates of catheter and surgical aortic arch reintervention (AAR) in this cohort. Timing of VCD diagnosis in relationship to other postoperative events and diagnoses is not gathered as part of this dataset.

Baseline characteristics were collected, including sex, birth weight, gestational age, primary cardiac diagnosis, and the presence of major syndromes or congenital anomalies. Major syndromes were defined as 22q11 deletion, CHARGE association, Down syndrome, Heterotaxy syndrome, Jacobsen syndrome, Turner syndrome, and VACTERL syndrome. Congenital anomalies included major abnormality of brain, gastrointestinal system, kidney/ureter/bladder, larynx/trachea/bronchus, lung, and/or spine. Preoperative use of mechanical ventilation, mechanical circulatory support, and the presence of a tracheostomy were documented.

Operative details were analyzed, including the type of surgical shunt used during the Norwood operation, cardiopulmonary bypass time (CPB time), aortic cross-clamp time (XC time), circulatory arrest time, and cerebral perfusion time (CPT). Postoperative variables included the duration of mechanical ventilation, need for reintubation, use of tube feeds, and the need for AAR performed at any time following the Norwood operation. Instances of postoperative tracheostomy and G-tube placement before their first birthday were recorded in relation to VCD**.**

### Statistical Analysis

Patient demographics and clinical characteristics were summarized using descriptive statistics. Initial analysis used the chi-squared test to assess the associations between categorical variables and the primary outcome. For continuous variables, appropriate parametric and non-parametric tests were used to compare the means or medians. We used a standard 2-sample t-test without assuming equal variances. Logistic regression analysis was performed to evaluate the association between VCD and other clinically significant variables, while adjusting for potential confounding variables including age and weight at time of Norwood, gender, presence of major congenital anomalies and syndromes, and type of surgical shunt at time of Norwood. The odds ratios (OR) are derived from logistic regression models to measure the likelihood of the primary outcome occurring in relation to predictor variables. The OR are provided with 95% confidence intervals and p-values to assess their statistical significance. For analysis of the relationship between hospital volume for Norwood operations per year or rates of AAR and VCD, the results are presented with estimated coefficients (Est), standard errors (SE), 95% confidence intervals (CI), and p-values, indicating the strength and significance of the associations. To illustrate the variation in VCD across different centers, a funnel plot was produced. Confidence intervals were computed using the Agresti-Coull method to account for variability in smaller sample sizes. Additionally, for each center, the proportion of patients who developed VCD was tabulated. These proportions were used to compare VCD rates and variability across different centers and to visualize how rates of VCD change over time. The analyses were done in SAS 9.4 and GAUSS 24.0.

## Results

**Fig. 1:**
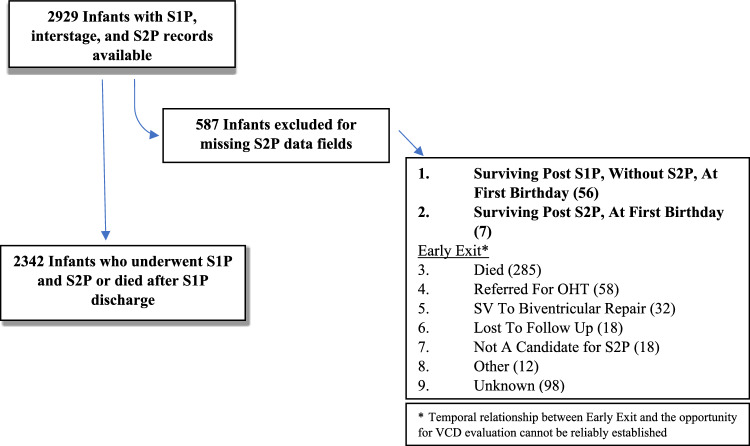
Flow diagram of participants

Of the 2,929 infants with S1P, interstage, and S2P records, 2,342 complete records were reviewed (Fig. 1). Clinical characteristics of this cohort are described in Table 1. Hypoplastic left heart syndrome was the most common diagnosis and most patients underwent the Norwood operation with a Sano shunt. VCD was diagnosed in 21.7% of patients, and most of those were diagnosed during the hospitalization immediately following the Norwood operation. Of the 2,093 infants who survived through S2P, 41 (2%) had VCD diagnoses recorded after S2P. AAR was performed in 19% of patients. Within the entire population, 611 (26%) required postoperative G-tube while the need for tracheostomy was much less common (3.3%). There were 4 other postoperative procedures recorded in S1P, 1 vocal cord (VC) medialization and 3 VC injections; 2 recorded during interstage, VC injections; and 3 recorded before 1 year birthday, 2 VC medializations and 1 VC injection.

**Table 1:** Clinical characteristics

	*N* (%)
Primary diagnosis (*N* = 2342)	
Hypoplastic left heart syndrome	1758 (75)
AA/MA	749 (43)
AA/MS	454 (26)
AS/MS	454 (26)
AS/MA	64 (3.6)
Other	35 (2)
Double inlet left ventricle	120 (5)
Unbalanced AV canal	104 (4)
DORV variant	91 (3.8)
Tricuspid atresia with TGA	87 (3.7)
Mitral atresia	35 (1.5)
Double inlet right ventricle	7 (0.3)
Other HLHS variant	140 (6)
Operative data	
Shunt type	
Sano	1605 68.5)
BTT	675 (28.8)
Central	62 (2.7)
Outcomes	
Survived through S2P hospitalization	2093 (94)
Vocal cord dysfunction	507 (21.7)
Diagnosed after stage 1	466 (92)
Diagnosed after stage 2	41 (8)
Aortic arch reintervention	456 (19)
Postoperative gastrostomy tube	611 (26)
Postoperative tracheostomy	79 (3.3)

Table 2 provides a comparison of characteristics of patients with and without VCD. Baseline characteristics were similar except for a slight Hispanic predominance in those with VCD. Patients with VCD have a comparable incidence of major syndromes and congenital anomalies to those without VCD. The distribution of shunt types at S1P showed no significant difference between the groups (*p* = 0.396). Regarding postoperative characteristics, average days of mechanical ventilation were similar between the groups, but respiratory failure requiring reintubation was more common in those with VCD (*p* = 0.001). Initial observations also revealed that there was a higher rate of AAR in patients with VCD compared to those without (25% vs. 18%, *p* = 0.001)*.* CPB time and XC time were both slightly longer in patients who developed VCD, whereas circulatory arrest time was shorter. Postoperative feeding methods included both tube and oral feeding but presence of G-tube at 1 year was much more common in those with VCD (42% vs. 22%, *p* < 0.001).

**Table 2:** Characteristics of patients with and without vocal cord dysfunction

Variable	No VCD *N* = 1835 (%)	VCD *N* = 507 (%)	*p* Value
Demographics			
Sex			
Male	1164 (63.3)	300 (59)	0.083
Race			0.186
White	1212 (69)	341 (69)	
Black	300 (17)	82 (17)	
Asian	22 (1)	8 (2)	
More than 1	47 (3)	5 (1)	
Other	179 (10)	59(12)	
Hispanic	296 (17)	103 (21)	0.031
Birth weight, mean kg (SD)	3.20 (0.57)	3.19 (0.59)	0.906
Gestational age, mean weeks (SD)	38.6 (1.35)	38.6 (1.24)	0.992
Major syndrome	169 (9)	52 (10)	0.476
Major congenital anomaly	100 (5)	33 (7)	0.362
Preoperative mechanical ventilation	331 (18)	84 (17)	0.443
Shunt at S1P			0.396
Sano	1245 (68)	360 (71)	
BTT	540 (29)	135 (27)	
Central	50 (3)	12 (2.6)	
CPB, mean min (SD)	160 (55)	170 (57)	< 0.001
XC, mean min (SD)	72.0 (30.9)	76.6 (30.1)	0.004
Circulatory arrest, mean min (SD)	22.5 (22.8)	18.4 (20.4)	0.002
CPT, mean min (SD)	65.9 (27.8)	65.6 (26.6)	0.880
Postoperative characteristics			
Days of mechanical ventilation, mean (SD)	18.4 (1.3)	17.8 (15.3)	0.460
Reintubation rates	370 (20)	136 (27)	0.001
Aortic arch reintervention	330 (18)	126 (25)	0.001
Postoperative tracheostomy	63 (3)	16 (3)	0.759
Postoperative G-tube placement	401 (22)	211 (42)	< 0.001

Multivariable logistic regression analysis using VCD as the primary outcome variable revealed that longer XC time remained a risk factor for development of VCD (Table 3). Meanwhile, shorter circulatory arrest time was a negative predictor after adjusting for other covariates. Reintubation during hospitalization after Norwood operation was associated with increased risk for VCD diagnosis (*p* = 0.002). AAR was associated with increased likelihood of VCD with OR of 1.525 (*p* = 0.001).

**Table 3:** Multivariable logistic regression for vocal cord dysfunction with various risk factors

	Odds ratio (95% CI)	*p* Value
Age at surgery	0.998 (0.974–1.022)	0.851
Female	1.179 (0.949–1.464)	0.137
Weight at S1P	1.067 (0.862–1.319)	0.553
Gestational age at birth	0.989 (0.708–1.382)	0.948
Presence of major syndrome	1.047 (0.728–1.505)	0.805
Presence of major congenital anomaly	1.255 (0.798–1.973)	0.326
Cardiopulmonary bypass time	1.002 (1.000–1.005)	0.075
Cerebral perfusion time	0.997 (0.994–1.001)	0.070
Circulatory arrest time	0.992 (0.986–0.998)	0.007
Cross clamp time	1.004 (1.000–1.008)	0.027
Norwood/BTT shunt	0.978 (0.492–1.944)	0.950
Norwood/sano	1.092 (0.558–2.137)	0.797
Days on mechanical ventilation	0.992 (0.985–0.999)	0.031
Reintubation	1.476 (1.153–1.888)	0.002
Aortic arch reintervention	1.525 (1.187–1.985)	0.001

**Fig. 2:**
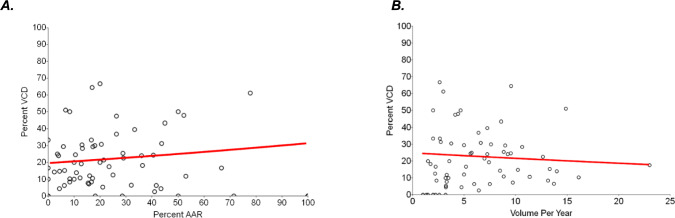
Vocal cord dysfunction rates in relation to AAR and hospital volume for Norwood. **A** Center-based analysis demonstrating positive correlation between rates of AAR and VCD. **B** A trend toward lower VCD rates at higher volume centers was noted

**Fig. 3:**
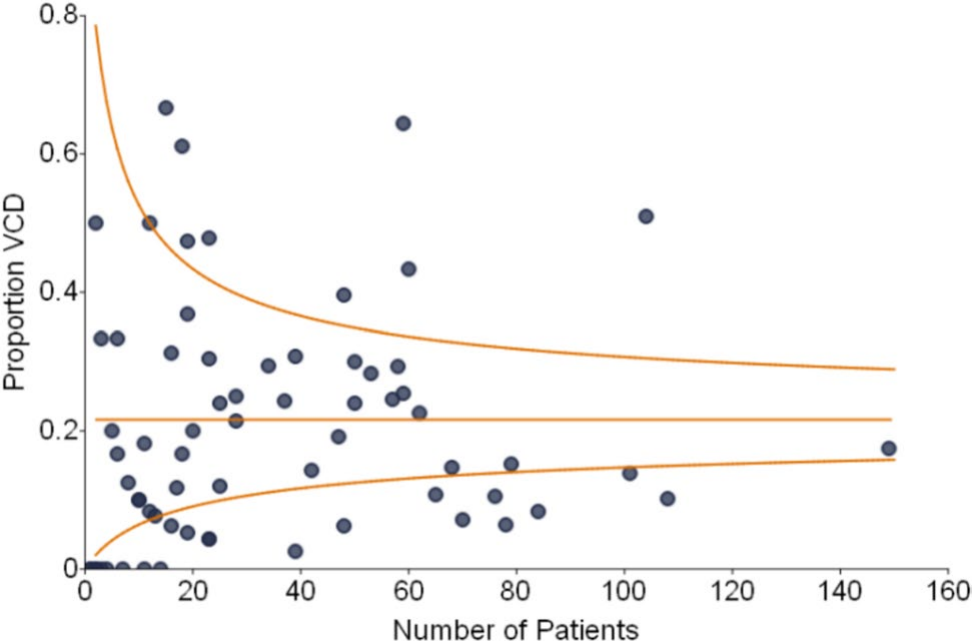
Center variation in rates of VCD. Center-based variability in VCD diagnosis rates after Norwood operation. Many centers fall within expected variation, but there are outliers above and below the control limits possibly reflecting inconsistent diagnostic practices

To explain this relationship further, Fig. 2A demonstrates a center-focused analysis of the association of AAR and VCD rates. Centers with higher AAR tend to also have higher VCD, however, this was not statistically significant (*p* = 0.059). When restricting to high procedure volume centers (≥ 10 AAR), this relationship became statistically significant (*p* = 0.008). The relationship between hospital volume for Norwood and diagnosis of VCD is illustrated in Fig. 2B. It revealed a negative association between the two, with lower rates of VCD diagnosis observed in higher volume centers (*p* = 0.072). However, when excluding one large-volume center, the relationship weakens significantly (*p* = 0.210). Figure 3 is a funnel plot depicting center variation for rate of VCD diagnoses, based on total number of Norwood operations at each center. There is considerable variability among centers. While rates of VCD at many centers remain within expected variation, at both high and low volume centers, there are clearly outliers above and below the control limits.

**Fig. 4:**
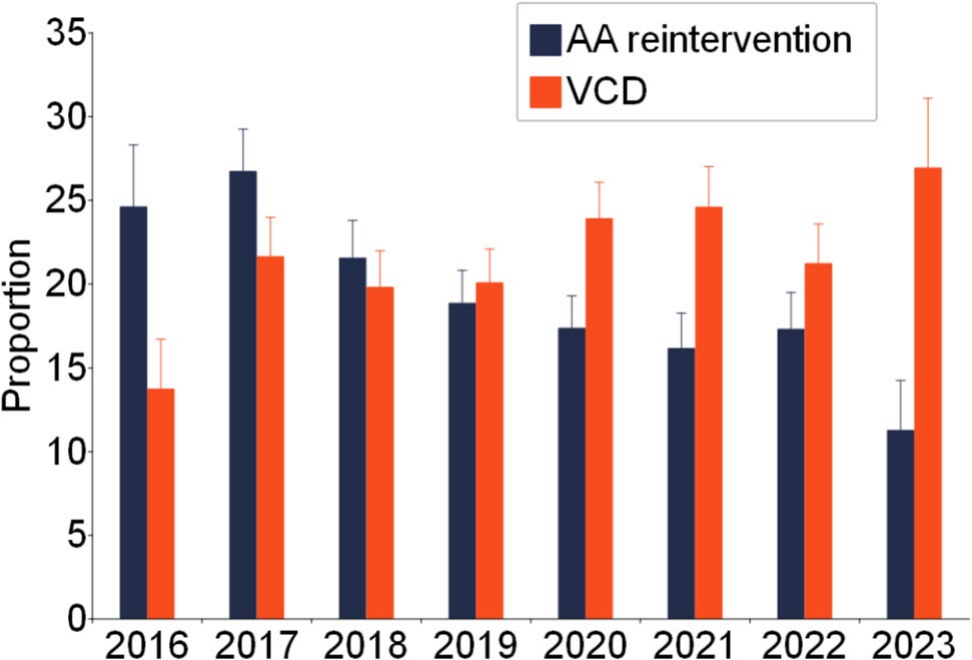
Changes in AAR and VCD over Time. AAR steadily declined over the study period while VCD generally increased, reflecting evolving surgical strategies, diagnostic awareness, or differences in institutional diagnostic practices

Given the previously described association between VCD and AAR, further analyses were performed to examine this relationship. Specifically, Fig. 4 demonstrates the temporal changes in rates of AAR and VCD over time, as determined by birth year. There seem to be contrasting trends between the two. Over the study period, AAR rates steadily declined, while VCD rates fluctuated but generally trended upward with the highest rates of diagnosis demonstrated in the most current year increasing from 13 to 26%.

## Discussion

Infants with HLHS and other single ventricle variants often require a series of surgeries involving aortic arch reconstruction. This requires extensive dissection and manipulation of important structures including the RLN which can lead to intraoperative injury resulting in vocal cord immobility. Using a large multicenter database, this study provides clearer insights into the prevalence of, risk factors for, and outcomes associated with VCD following the Norwood operation. Moreover, center variation for VCD diagnosis was explored.

Prior studies report a wide range in incidence of VCD which is likely due to institutional differences in surgical volume and lack of consistent approach to diagnosis of VCD including methods of and threshold for diagnosis [1, 2, 6, 10, 11]. In this cohort, we found that VCD was diagnosed in nearly 22% of infants, falling in between the reported estimates from single-center studies on infants undergoing cardiac surgery including aortic arch operations. Higher counts tend to come from single-center studies where routine laryngopharyngeal evaluation after Norwood operation or similar aortic arch operations is common [1]. Whereas the lower range of 1.1% is reported from studies evaluating VCD after any congenital cardiac surgery [2]. It’s crucial to recall that VCD was diagnosed based on endoscopic evaluation only. Other modalities of diagnoses, especially laryngeal ultrasounds, were uncommonly mentioned but could not be linked to VCD in this cohort.

Longer CPB and XC times were identified as risk factors for VCD on both adjusted and unadjusted analysis, suggesting that prolonged surgical intervention increases the risk of iatrogenic injury to the RLN. In the univariate analysis, the difference in times was quantitatively very small and likely due to our large sample size which allowed us to detect small differences. Regardless, these differences may not be clinically meaningful. There was a higher rate of VCD diagnosis in infants who underwent AAR, however, it is difficult to establish causality. Since timing of VCD cannot be determined in relation to AAR, it’s unclear if VCD developed before or after AAR during the same hospitalization. Similarly, reintubation was associated with an increased likelihood of VCD diagnosis. While RLN neuropraxia from endotracheal intubation has been recognized as an etiology for VCD [8], reintubation itself could also be a proxy indicator of RLN injury sustained during hospitalization after index operation. Alternatively, it’s important to consider heightened suspicion for VCD in an infant who required reintubation, potentially leading to more diagnoses made and explaining this association. Again, the lack of temporal association with VCD diagnosis makes this difficult to thoroughly evaluate in this study.

When looking at trends over time, the rate of VCD diagnosis increased over the study period potentially suggesting advancements in diagnostic methods and an evolving understanding of VCD [12]. Improved diagnostic ability using both DFL and FEES and heightened awareness may result in more cases identified. Rates of AAR have declined over the same period. It is possible that a trend towards more extensive arch reconstruction during the index operation has led to lower rates of AAR at the behest of higher rates of VCD. On the other hand, rates of AAR and VCD diagnosis were positively correlated at both the individual patient and center-level. This association can be explained in multiple ways. Multiple surgeries on the arch can certainly increase the risk of RLN injury, leading to true increase in rates of VCD. It could be argued that evaluation of vocal cord function may be done more frequently in patients who undergo AAR, leading to increased diagnosis of VCD in this group even if rates are not truly higher than in patients who do not undergo AAR. Alternatively, patients who underwent AAR and had a diagnosis of VCD may be sicker and more complex.We attempted to control for some patient factors that reflect this, but unmeasured variables may be confounders. Not knowing the timing of VCD diagnosis, makes it challenging to interpret the relationship between AAR and VCD. Therefore, a causal relationship should not be inferred as accurate dating was not available for review.

In this NPC-QIC cohort, there is considerable variability in VCD diagnosis rates across surgical centers. A total of 67 centers from the registry were used in the analysis and participation varied. Some centers joined or left the collaborative at different times. Generally, higher volume centers demonstrated lower rates of diagnosis of VCD. Differences in evaluating vocal cord function also vary by center including thresholds for evaluation (screening all patients versus only those with symptoms) and by method of evaluation. How those diagnoses are reported are likely to differ as well. It is difficult to determine if this relationship reflects differences in surveillance for VCD versus differences in true diagnosis of VCD. Patients with VCD by evaluation but without clinical signs would be more likely to be found at centers who routinely evaluate vocal cord function in all or most patients compared to centers which only evaluate when there are clinical signs.

An inverse relationship between hospital volume and in-hospital mortality has been reported, especially in those who already carry a high surgical risk-profile [13]. Even in the recent era, infants who undergo the Norwood operation experience more postoperative complications and have higher odds of mortality at lower volume centers [14]. Center variation could be due to institutional differences in patient characteristics, surgical techniques as well as institutional practices regarding diagnostic thresholds and methods [9]. It has also been suggested that the recognition of complications and the effective management of those complications by experienced personnel are optimized at higher volume centers [14]. Further investigation is likely needed to determine what factors influence this.

The potential effects of VCD on swallowing and respiratory functions warrant further attention. Infants with VCD are more commonly confronted with feeding challenges and need for non-cardiac operations. Those affected have increased need for tube feeding, up to 46%, compared to their cohorts without VCD [1]. The need for tube feeding often persists at time of discharge and one study reports that 55% need G-tube placement [5]. Non-cardiac interventions, such as tracheostomy, vocal fold injections, and thyroplasty, are also common in this group of infants. In alignment with prior literature, this cohort of infants with VCD also have feeding difficulties and need prolonged enteral feeding support. The high rates of G-tube placement in patients with VCD (42% in patients with VCD vs. 22% without) highlights its potential role in supporting postoperative care and maintain quality of life in patients and their caregivers. Alternatively, tracheostomy rates were relatively low (3.3%), suggesting that even though VCD is common, airway compromise requiring surgical intervention is less frequent in patients that are able to be discharged. While recovery rates vary across studies, VCD can persist in as many as 65% of patients nearly a year after diagnosis [15]. Our findings emphasize the importance of identifying and mitigating risk factors for VCD after the Norwood operation.

## Limitations

There are several limitations to acknowledge. First, the retrospective nature and use of a data registry introduces both selection and reporting biases. Inherent limitations of a database include incomplete data fields or unmeasured variables in the original data form as well as diverse composition of patients from the participating programs**.** Regarding our study design, combining patients who survived to S2P with those who died after S1P introduces differing times at risk for VCD. We did this to maximize our sample size within the limitations of the dataset. In those who exited the study before S2P, it is also possible that they had meaningful differences from those who remained within the dataset. Operative details and timing of VCD diagnosis were not recorded at the onset, making it difficult to draw casual inferences or explain certain associations especially in relation to AAR. As noted above, there is a lack of standardized evaluation for diagnosis or VCD which affects his prevalence and how it compares between institutions. While some center routinely use endoscopy for screening, other rely solely on clinical diagnosis. Furthermore, excluding those with missing data may underestimate or overestimate the true prevalence of VCD in this cohort. Analysis of the center variation was limited to rates of VCD diagnosis by volume and did not control for other clinical factors or center practices. Future studies could consider a multicenter prospective design with standardized diagnostic protocols including endoscopic and ultrasound evaluation of vocal cord mobility to verify these findings and identify additional risk factors.

## Conclusion

This study reports on the prevalence of and possible clinical impact of VCD in high-risk infants undergoing the Norwood operation. Moreover, center variability in diagnostic rates and methods is undeniable. It may be prudent to standardize diagnostic protocols, management strategies, and enhance multidisciplinary care given its significant clinical burden.

## Data Availability

No datasets were generated or analysed during the current study.

## References

[1] Averin K, Uzark K, Beekman RH, Willging JP, Pratt J, Manning PB (2012) Postoperative assessment of laryngopharyngeal dysfunction in neonates after Norwood operation. Ann Thorac Surg 94(4):1257–1261. 10.1016/j.athoracsur.2012.01.009 22421593 10.1016/j.athoracsur.2012.01.009

[2] Alfares FA, Hynes CF, Ansari G, Chounoune R, Ramadan M, Shaughnessy C et al (2016) Outcomes of recurrent laryngeal nerve injury following congenital heart surgery: a contemporary experience. J Saudi Heart Assoc 28(1):1–6. 10.1016/j.jsha.2015.05.00 26778899 10.1016/j.jsha.2015.05.002 PMC4685232

[3] Gorantla SC, Chan T, Shen I, Wilkes J, Bratton SL (2019) Current epidemiology of vocal cord dysfunction after congenital heart surgery in young infants. Pediatr Crit Care Med 20(9):817. 10.1097/PCC.0000000000002010 31246739 10.1097/PCC.0000000000002010

[4] Sachdeva R, Hussain E, Moss MM, Schmitz ML, Ray RM, Imamura M et al (2007) Vocal cord dysfunction and feeding difficulties after pediatric cardiovascular surgery. J Pediatr 151(3):312-315.e2. 10.1016/j.jpeds.2007.03.014 17719946 10.1016/j.jpeds.2007.03.014

[5] Truong MT, Messner AH, Kerschner JE, Scholes M, Wong-Dominguez J, Milczuk HA et al (2007) Pediatric vocal fold paralysis after cardiac surgery: rate of recovery and sequelae. Otolaryngol Neck Surg 137(5):780–784. 10.1016/j.otohns.2007.07.028 10.1016/j.otohns.2007.07.028 17967646

[6] Pourmoghadam K, DeCampli W, Ruzmetov M, Kosko J, Kishawi S, O’Brien M et al (2017) Recurrent laryngeal nerve injury and swallowing dysfunction in neonatal aortic arch repair. Ann Thorac Surg 1:104. 10.1016/j.athoracsur.2017.03.080 10.1016/j.athoracsur.2017.03.080 28648533

[7] Raut MS, Maheshwari A, Joshi R, Joshi R, Dubey S, Shivnani G et al (2016) Vocal cord paralysis after cardiac surgery and interventions: a review of possible etiologies. J Cardiothorac Vasc Anesth 30(6):1661–1667. 10.1053/j.jvca.2016.08.002 27769765 10.1053/j.jvca.2016.08.002

[8] Evman MD, Selcuk AA (2020) Vocal cord paralysis as a complication of endotracheal intubation. J Craniofac Surg 31(2):e119. 10.1097/SCS.0000000000005959 31985591 10.1097/SCS.0000000000005959

[9] Narawane A, Rappazzo C, Hawney J, Clason H, Roddy DJ, Ongkasuwan J (2022) Vocal fold movement and silent aspiration after congenital heart surgery. Laryngoscope 132(3):701–705. 10.1002/lary.29817 34378798 10.1002/lary.29817

[10] Khariwala SS, Lee WT, Koltai PJ (2005) Laryngotracheal consequences of pediatric cardiac surgery. Arch Otolaryngol Neck Surg 131(4):336. 10.1001/archotol.131.4.336 10.1001/archotol.131.4.336 15837903

[11] Pereira KD, Webb BD, Blakely ML, Cox CS, Lally KP (2006) Sequelae of recurrent laryngeal nerve injury after patent ductus arteriosus ligation. Int J Pediatr Otorhinolaryngol 70(9):1609–1612. 10.1016/j.ijporl.2006.05.001 16797086 10.1016/j.ijporl.2006.05.001

[12] Langmore SE (2017) History of fiberoptic endoscopic evaluation of swallowing for evaluation and management of pharyngeal dysphagia: changes over the years. Dysphagia 32(1):27–38. 10.1007/s00455-016-9775-x 28101663 10.1007/s00455-016-9775-x

[13] Pasquali SK, Li JS, Burstein DS, Sheng S, O’Brien SM, Jacobs ML et al (2012) Association of center volume with mortality and complications in pediatric heart surgery. Pediatrics 129(2):e370–e376. 10.1542/peds.2011-1188 22232310 10.1542/peds.2011-1188 PMC3269112

[14] Welke KF, Karamlou T, O’Brien SM, Dearani JA, Tweddell JS, Kumar SR et al (2023) Contemporary relationship between hospital volume and outcomes in congenital heart surgery. Ann Thorac Surg 116(6):1233–1239. 10.1016/j.athoracsur.2023.08.006 37652353 10.1016/j.athoracsur.2023.08.006

[15] Pham V, Connelly D, Wei JL, Sykes KJ, O’Brien J (2014) Vocal cord paralysis and dysphagia after aortic arch reconstruction and Norwood procedure. Otolaryngol Neck Surg 150(5):827–833. 10.1177/0194599814522413 10.1177/0194599814522413 PMC4262533 24515967

